# Clinic

**Published:** 1907-11

**Authors:** F. H. Houghton


### CLINIC.

By F. H. Houghton, D. D. S.

Demonstrating the use of the rubber dam, for operation on the teeth in a comparatively painless way, without ligating the teeth.

Of all the operations the dentist performs for his patient, relating to operations in dentistry, there is none in my judgment so important, as carefully and considerately adjusting the rubber dam.

Our patients come to us, as a rule, under protest, they expect to be hurt, and so put off the dreaded day until the offending tooth drives them to the executioner.

My experience of many years has taught me that one of the most dreaded of all operations, by our patients, is having the rubber dam placed and tied around their teeth. And, gentlemen, while it is our duty to make all operations as painless as possible for our patients, it applies especially to adjusting the rubber dam, and so it is our duty to make the operation in the most careful, humane, tender and considerate way possible.

There is no one but who appreciate kindly treatment at the hands of a dentist, besides it is "casting bread upon the waters;" the reflex is far reaching and for obvious reasons accrues to the best interest of the dentist.

Adjusting the rubber dam without ligating the teeth has been my practice for more than twenty years, and I find it a rare exception when necessary to ligate a tooth.

The purpose of the dam is first to keep the tooth dry during the operation, a condition that is imperative for a successful operation. Secondly, to give us all possible control and advantage of the operation, for instance we have cavities in the upper central and lateral teeth, suppose the teeth are all in place, we first make eight openings in the dam about ¼ of an inch apart, this gives you the first bicuspids the natural shapes of which hold the dam in position, in most instances, without further retainer, in case it does not, gently place a clamp to hold in place, to do this it is not necessary to impinge the gum tissues in the least. Including these eight teeth in affixing the dam, we first get a good lighting of all the teeth, secondly we have plenty of room to handle our instrument and giving us all and every advantage over the operation.

Now in the words of the lamented Davy Crocket we repeat "be sure you are right and then go ahead." So we first take a small silk thread or preferably buttonhole twist, well waxed, pass it carefully between the teeth to make sure there is no obstacle to prevent the passing of the dam between the teeth. In making this preparation should we find an impediment such as a sharp edge of a cavity to cut the dam or at the shoulder line a crowding or binding together of the teeth; take some one of the many thin saws of files and, by gentle manipulation, dispose of the obstacle. Now we have a clear way for placing the dam. Looking well after this preliminary feature, will save, many times, needless worry and vexation of spirit.

We are now ready to place the dam. Begin by enveloping the tooth that presents the best form and conditions for holding the dam. Manipulating with the fingers in a gentle way by holding toward one side of the opening in the dam, now with a little lateral working or sliding motion it goes easily between the teeth. Should it become necessary to use the silk do so by holding same, well waxed, between thumb and forefinger of each hand holding same taut, with a lateral sliding motion you carry the dam gently to place, never put a hard strain on silk toward neck of tooth or gum line for fear of suddenly striking and injuring the gums.

Now we carry the dam neatly to place about the neck of the teeth take silk thread or twist pass some over and under lingual side of tooth, holding the two ends of silk firmly, between thumb and fingers of left hand, on buccal surface of tooth we now take a straight smooth burnisher and gently push the silk to the gum line which carries the dam with it, a little gentle manipulating with the smooth burnisher will complete the placing of the dam and it will be most satisfactory to your patient and comparatively painless, but only at time of operation but at no subsequent time will patient suffer from sore or tender gums. Dam is less liable to be ruptured by wheels, burs, or instrument of any kind and in case such accident does happen the trouble is remedied in much less time and without pain to your patient, than if tied on in the good old hurting way. Remove your silk or twist, the rubber will take care of itself and when you remove rubber from the teeth you will find it much more securely in place then when tied on with ligatures. Your patient will leave your office with an appreciative heart and tell the first friend she meets how gentle and kind you were and that you did not hurt in adjusting that horrid rubber dam. Try it.

The rubber dam in the hands of a careful dentist is a boon to both patient and operator. God bless Mr. Barnum the inventor of the rubber dam, who so generously and freely gave it to suffering humanity, without money and without price. His name should be engrossed in a hallow of glory and be perpetuated forever.

